# S1. THE ASSOCIATION BETWEEN WAR-RELATED STRESS, PTSD SYMPTOMS, AND SUB-CLINICAL PSYCHOSIS: A CROSS-CULTURAL POPULATION-BASED STUDY AMONG PALESTINIAN AND ISRAELI YOUNG ADULTS

**DOI:** 10.1093/schbul/sby018.788

**Published:** 2018-04-01

**Authors:** Amjad Mussa, Meirav Blizovski, Dan Koren

**Affiliations:** University of Haifa

## Abstract

**Background:**

Sub-clinical or attenuated psychosis symptoms (APS) in the general population has become a focus of considerable research interest over the past two decades, as they appear to index an increased risk for psychotic outcomes. Recent data from several community-based studies around the world provide convincing support for an association of APS with traumatic stress that is likely moderated by familial genetic risk and gender. However, relatively little is known about the degree to which APS are associated with terror/war-related stress. Moreover, relatively little is known about the degree to which cultural/religious factors moderate this association. Hence, the overarching goal of this study was to address this lacuna in the literature by examining the relationship between exposure to terror/war-related events, PTSD symptoms and familial genetic risk among Palestinian and Israeli youth.

**Methods:**

Exposure to terror/war-related trauma, presence and severity of PTSD symptoms, perceived ability to cope with trauma, familial genetic-risk, and APS were assessed in a representative sample of 530 Israeli and 1100 Palestinian (451 from Israel, 264 from the West Bank, and 385 from the Gaza Strip) young adults with a mean age of 36.7 (SD=8.4). PTSD symptoms were assessed with the Post-traumatic Disorder Scale (PDS), perceived ability to cope with trauma with the Perceived Ability To Cope With Trauma Scale (PACT), and APS with the Community Assessments of Psychic Experiences (CAPE).

**Results:**

As hypothesized, there was a significant three-way interaction effect of exposure to terror-war-related trauma, religion, and familial genetic-risk on APS. The highest level of APS was among Palestinians who live in the Gaza strip, with no significant differences between Jews and Palestinians who live in Israel or in the West Bank. Also, consistent with our hypotheses, the three-way association between exposure to trauma, familial genetic risk and religion was mediated by PTSD symptoms and perceived ability to cope with trauma.

**Discussion:**

These findings provide further support for the link between exposure to trauma, familial genetic-risk, and APS. Also, it provides further support for the mediating role that PTSD symptoms play in this link. Finally, it suggests that religious background moderates the link between exposure to trauma and APS.

